# Investigation of Weigh-in-Motion Measurement Accuracy on the Basis of Steering Axle Load Spectra

**DOI:** 10.3390/s19153272

**Published:** 2019-07-25

**Authors:** Dawid Rys

**Affiliations:** Department of Highway and Transportation Engineering, Faculty of Civil and Environmental Engineering, Gdansk University of Technology, 80-263 Gdansk, Poland; dawid.rys@pg.edu.pl

**Keywords:** weigh-in-motion, overweight vehicles, overloaded vehicles, heavy traffic, axle load spectra, steering axle, bending beam, piezoelectric, piezoquartz, axle load sensors

## Abstract

Weigh-in-motion systems are installed in pavements or on bridges to identify and reduce the number of overloaded vehicles and minimise their adverse effect on road infrastructure. Moreover, the collected traffic data are used to obtain axle load characteristics, which are very useful in road infrastructure design. Practical application of data from weigh-in-motion has become more common recently, which calls for adequate attention to data quality. This issue is addressed in the presented paper. The aim of the article is to investigate the accuracy of 77 operative weigh-in-motion stations by analysing steering axle load spectra. The proposed methodology and analysis enabled the identification of scale and source of errors that occur in measurements delivered from weigh-in-motion systems. For this purpose, selected factors were investigated, including the type of axle load sensor, air temperature and vehicle speed. The results of the analysis indicated the obvious effect of the axle load sensor type on the measurement results. It was noted that systematic error increases during winter, causing underestimation of axle loads by 5% to 10% for quartz piezoelectric and bending beam load sensors, respectively. A deterioration of system accuracy is also visible when vehicle speed decreases to 30 km/h. For 25% to 35% of cases, depending on the type of sensor, random error increases for lower speeds, while it remains at a constant level at higher speeds. The analysis also delivered a standard steering axle load distribution, which can have practical meaning in the improvement of weigh-in-motion accuracy and traffic data quality.

## 1. Introduction

### 1.1. Background

Weigh-in-motion (WIM) systems are installed in pavements or on bridges for two main purposes: (1) to identify and reduce the number of overloaded vehicles and minimise their adverse effect on road infrastructure [1,2,3,4,5,6,7,8]; and (2) to collect traffic data, especially axle load characteristics [9,10,11,12,13,14,15]. Application of actual data from WIM for pavement distress analysis results in more reliable estimations of maintenance treatment schedule and agency costs [16]. Use of WIM data in pavement analysis requires due attention to data quality. Efforts to improve WIM data quality are laid both on the development of new solutions for WIM construction, like multi-sensors systems [17,18], and in developing new procedures for data processing [19]. The significance of data quality in pavement design was studied in [16,20,21,22], Farkhideh and Nassiri [20] investigated weight measurements from WIM systems installed in asphalt concrete pavements. They reported that WIM errors were reflected in the axle load characteristics for design according to the Mechanistic-Empirical Pavement Design Guide (MEPDG), and they did affect the thickness of the designed pavement structure—in some cases by more than 100%. Haider et al. [21,23] investigated the effect of change in systematic error for a given random error on both flexible and rigid pavement design and concluded that a 10% positive bias leads to overestimation of pavement life by approximately 5%, while in contrast, a 10% negative bias will result in underestimation of pavement life by approximately 30% to 40%. Prozi and Hong [22] reached similar conclusions in their work, whose results suggested that load-pavement impact estimation error is more sensitive to over-calibration than under-calibration of the WIM system. The aforementioned findings prove the significance of WIM system accuracy when the statistical data are further used for pavement design and analysis.

Due to the significance of data quality in pavement design, relevant experience was collected and described in the NCHRP Report no. 538 [24], FHWA-IF-10-018 report [25] and Cost 323 specification [26]. According to these works, the primary sources of WIM inaccuracy lie in invalid vehicle detection and classification [27] as well as in the uncertainty of axle load measurements. While specific sets of algorithms [28,29] enable identification of incorrectly recognised vehicles and their elimination from the database, the problem of validation of axle load measurements is more complex. According to the ASTM E1318 standard [30] and Cost 323 specification [26], the accuracy of a given WIM system in terms of axle loads is expressed as the relative difference between WIM weight measurements and those from a static scale. To ensure the required accuracy level, WIM systems are calibrated using standard procedures that compare measurements obtained from WIM and from static scales. It is noteworthy that WIM system accuracy is not described exclusively by the accuracy of the axle load sensor but by the combined properties of pavement and sensor. Regardless of WIM calibration, which ensures the required accuracy of the system for a limited period, the accuracy deteriorates over time due to several factors, including: (1) changes in measurement conditions (e.g., temperature) [31]; (2) increase in pavement deflections and roughness resulting from pavement distress [32]; and (3) fatigue of load sensors [33,34]. The tendency of WIM sensors to lose calibration over time was described in greater detail in a study by Chatterjee et al. [35].

More advanced procedures of WIM data validation were proposed by Mai, Turochy and Tim [28]. The previous works describe methods of identification of inaccurate WIM data, which should be removed from further analysis. A study conducted by Nichols et al. [36] as well as a further study by Burnell et al. [19] proposed WIM accuracy evaluation based on left-right wheel weight measurement difference for each axle. They reported that the steering (front) axle in articulated vehicles with semi-trailer (class 9 according to FHWA vehicle classification) tends to have a much lower variance in comparison to other axles and its load depends on gross vehicle weight (GVW) to a lesser degree, which was also confirmed in the studies of Burnos and Gajda [31].

The aim of the presented paper is to contribute to the improvement of WIM system accuracy by undertaking an investigation of steering axle load spectra. The proposed methodology and results of the study can be helpful in: (1) validation and improvement of data quality used for pavement design and analysis; and (2) development of algorithms for self-calibration of WIM systems to improve their accuracy.

### 1.2. Objective and Scope

The main objective of the paper is to investigate steering axle load distributions derived from a large database of 77 WIM stations operating in normal service in Poland. The aim of the proposed methodology and analysis is to identify the scale and sources of potential errors that may occur in measurements delivered from WIM systems. For this purpose, selected factors which have an impact on the accuracy of WIM systems were investigated, including the type of axle load sensor, air temperature and vehicle speed. Moreover, the analysis delivered a standard steering axle load distribution, which can have practical meaning in the improvement of WIM accuracy and data quality.

## 2. Methodology

### 2.1. Effect of Weigh-in-Motion System Inaccuracy on Axle Load Spectra

Axle load errors are often expressed by relative difference between static and instantaneous dynamic load [21,22,37]. The relative error of a given measurement *δ*_*i*_ can be expressed by the following formula:

(1) $$\delta_{i}=\frac{w_{i}-w_{i,ref}}{w_{i,ref}}$$

where: *w_i_*—weighing result of the *i*-th axle obtained from the tested WIM system; *w_i,ref_*—reference result of weighing of the *i*-th axle on static scales.

The average value of relative errors obtained for a given number of observations is commonly referred to as the measurement error for a WIM scale and data depends on its accuracy. The relative error consists of two components: systematic and random error. In the case of systematic error, also referred to as bias in the literature, each value of measured load is shifted in relation to the real value of load. The main reason for the occurrence of systematic error is a change in measurement conditions in relation to the reference conditions that were used during calibration. Random errors arise from fluctuations in measurement, both positive and negative, from the real value. The main factors that have impact both on relative and random error are connected with dynamic loads from the moving vehicles (pavement smoothness, suspension and tyre characteristics, vehicle speed), imperfection of measuring instruments (load sensors and signal computing) and variations in measuring conditions (weather conditions, pavement material properties etc.).

**Figure 1:**
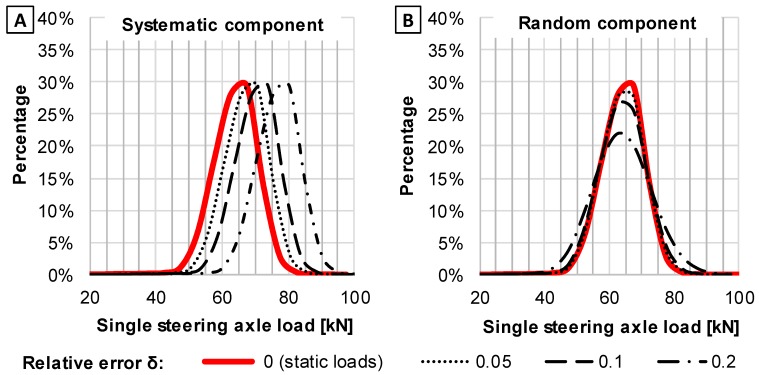
An example of the effect of (**A**) systematic and (**B**) random components of relative error on axle load spectrum (ALS).

Axle load spectra (further abbreviated to ALS) are calculated on the basis of the number of axles falling within a given interval of axle load. Due to errors of measurements, some axles are assigned to invalid load intervals. In consequence, ALS determined on the basis of weigh-in-motion data vary from ALS determined without error that occurs in WIM systems. The effect of WIM error on ALS is considered with regard to two error components: systematic and random errors. Figure 1 illustrates the effect of the systematic and random components of error on an example ALS. The WIM relative errors presented in Figure 1 were calculated according to Equation (1). In the considered example, the distribution with relative error δ = 0 corresponds to loads obtained from static scales. To illustrate the ALS and its change due to error occurrence, 1000 records of steering axle loads for 5 axle trucks with semi-trailers were chosen from the dataset described later in this paper. In order to obtain the ALS with a given error of measurement, each of the records were modified. The systematic error arises from the calibration bias. It means that when systematic errors occur, each of recorded loads fall into values shifted in comparison to real values. As a consequence, the whole ALS is shifted into what is illustrated in Figure 1A. When only random component errors occur, each of the recorded loads fall randomly with the normal distribution into values beyond the static load values. Consequently, the ALS becomes more “flatted”, what is illustrated in Figure 1B. The effect of systematic and random errors on the ALS shown in Figure 1 is in accordance with previous works [22].

### 2.2. Identification of Factors Responsible for WIM Inaccuracy on the Basis of Steering Axle Load Spectrum (SALS) Analysis

Steering axle loads can be used for the evaluation of WIM system accuracy [19,32,37]. This approach was used to identify factors that have the greatest impact on the accuracy of operative WIM systems as well as to estimate the scale of the problem. For this purpose, a group of commercial heavy vehicles was considered that is relatively uniform in terms of construction properties and very popular as well. Based on previous works [15,38], the best example of such a vehicle in Polish conditions is a five-axle articulated vehicle consisting of a tractor with two single axles (steering and drive) and a semi-trailer with one tridem axle. The gross weight of an empty vehicle ranges from approximately 16 to 20 tonnes (Mg). Construction of this vehicle results in a minor effect of semi-trailer kerb weight, freight weight and freight distribution on steering (first) axle load. This group of vehicles is very universal in terms of the type of freight it may carry. Therefore, it is expected that the steering axle load spectrum (further abbreviated to SALS) of the selected vehicle type shall be the same, regardless of traffic volume and character of transport on a given road. Moreover, any variations in traffic volume, speed or seasonal factors, including temperature, should have no impact on SALS.

As further analysis shows, SALS can be characterised with normal distributions, thus the mean value and standard deviation deliver a full description of SALS. If any relative errors occur, the deviations in the first axle load would result from random differences in the kerb weight of vehicles. Theoretically, it is expected that the mean and standard deviation of SALS should be constant, regardless of the localisation and measurement conditions of the station. Nevertheless, differences in the mean and standard deviation are observed and they indicate the inaccuracy of WIM systems. Differences in mean values reflect systematic error of load measurement. Differences in standard deviation imply random error.

**Figure 2:**
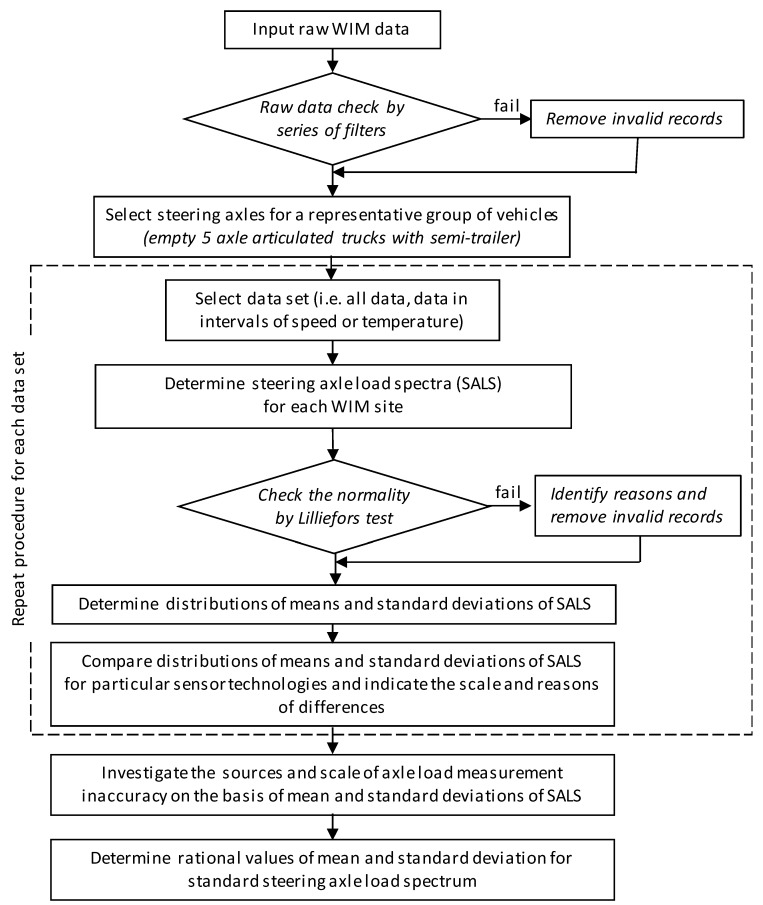
Scheme of the steps performed in the analysis to investigate the scale and sources of errors on weigh-in-motion (WIM) stations and to determine the standard steering axle load spectrum.

Figure 2 illustrates overall steps in the analysis which were performed to identify the scale and sources of potential errors that may occur in measurements delivered from WIM systems, and to deliver the standard steering axle load. The proposed methodology of determination of standard steering axle load is a step forward into new procedures of self-calibration algorithms in real time, which are a novelty in comparison to previous calibration procedures, and which are based on comparison of the relative difference between WIM and static weight measurements [24,25,26].

The steps performed in the analysis presented in Figure 2 can be explained as follows. At the first step, data from various 77 WIM stations were collected. The paper investigates various cases of SALS calculated from data selected using a series of filters. The first stage of the filtering process was focused on identifying and removing invalid raw records from the database. Vehicle records were removed when: the sum of the axle load was not equal to gross weight, any gap between neighbouring axles was lower than 0.5 m, vehicle length was lower than 3 m or greater than 20 m, the record was uncompleted or flagged as incorrect by the WIM system. The aim of the third step was to select steering axles for a group of vehicles represented by two-single-axle trucks with semi-trailer with tridem axle. In order to eliminate the effect of freight load and distribution, only empty vehicles with gross weights from 16 to 20 Mg were selected. 

In the further step remaining filters were selected depending on the following factors that were investigated in the study: type of axle load sensor, vehicle speed and air temperature. As previous studies show, pavement deflections (which in the case of asphalt pavements—are a function of load), pavement temperature and vehicle speed significantly impact the measurement obtained from the WIM load sensor [32]. Due to this fact, the intervals of filters set for vehicle speed and temperature were narrow and equalled 10 km/h and 5 °C, respectively. When filters are set, a large number of measurements is required to correctly determine ALS, therefore a wide range of data covering the whole year were used. The ranges of intervals were chosen as a compromise between the change of asphalt mixtures properties and number of records to represent reasonable measuring data from at least 1000 records. For the selected data, statistical analysis of the results obtained for particular WIM stations was further performed. Firstly, the SALS were calculated. When the Lillierfors test confirmed the normality of SALS then the mean values and standard deviations of SALS obtained from various stations at the same measurement conditions (air temperature, vehicle speed) were compared with the use of cumulative distributions charts. This comparison allowed us to imply differences in measurement results and potential random and systematic errors for two main axle load sensor technologies—bending beam and piezoquartz, and allowed us to imply how temperature and vehicle speed contribute to increasing these errors. The final output of the statistical analysis is a standard steering axle load spectrum which correspond to real traffic condition, regardless of sensor type, pavement structure, thermal conditions and vehicle speed.

## 3. Data Used in the Analysis

**Figure 3:**
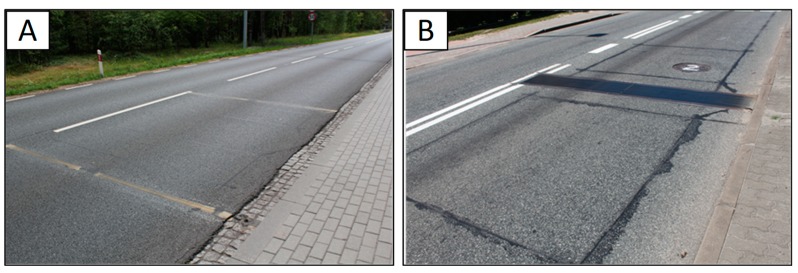
The investigated technologies of WIM axle load sensors: (**A**) quartz piezoelectric (piezoquartz); (**B**) bending plate.

The data considered in this study were collected from 77 WIM stations localised on motorways and national roads in Poland. The period of measurements was from 1 January to 31 December 2014. Table 1 includes a summary of information about the WIM stations. Stations were installed by five companies specialised in intelligent transport systems. Each station is equipped with axle load sensors based on one of the two technologies: quartz piezoelectric (54 stations) and bending beam (23 stations). Examples of load sensors of the two types installed in the pavement surface are presented in Figure 3.

The WIM systems used currently are not equipped with air or pavement temperature sensors. Hence, to provide information on temperature during measurements, the WIM data were supplemented by meteorological data delivered from weather stations administrated by the Polish Institute of Meteorology and Water Management (IMGW). Each measurement record for a vehicle weighted in motion was supplemented with air temperature measured at the same time in the nearest weather station. Air temperature is not equal to pavement temperature, nevertheless, they are strongly correlated. More detailed information about the application of air temperature data from Polish IMGW weather stations for calculations of pavement temperatures is included in [38,39,40,41]. For the purpose of this paper air temperatures are sufficient to represent the overall thermal conditions in the WIM station.

## 4. Results and Discussion

### 4.1. Determination of Steering Axle Load Spectra (SALS) and Normality Check

**Figure 4:**
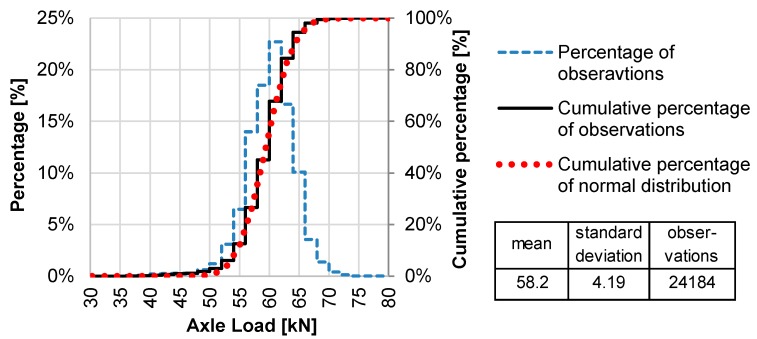
Example of steering axle load spectrum (SALS) determined for WIM station no. 58.

SALS were determined for each of the 77 WIM stations. SALS for WIM station no. 58 is presented in Figure 4 as an example. For each station the shape of SALS probability distributions is very close to normal distribution, which is also visible in the example given in Figure 4.

SALS were investigated in terms of normality of distribution. For this purpose, during the first stage, the Lilliefors test was conducted at the level of 99.9%. The results of the tests indicated that for 40% of the calculated distributions (including station no. 58 shown in Figure 4) the null hypothesis about normality of distribution can be rejected. In other words, only 60% of SALS meet the assumption that they are normal distributions. The problem with normality of distribution detected for 40% of stations resulted from the fact that, for these stations, a number of steering axles with loads below 40 kN were improperly detected as steering axles of a heavy articulated vehicle, while in fact they were axles of lighter vehicles, moving very close to heavier ones. This is probably due to an inaccuracy in the automatic vehicle classification module. While the proportion of inaccurately recognised vehicles was below 2% of the total number of axles, due to the strength of the Lilliefors test and a large number of observations, it had an impact on the test results. To eliminate this incoherence, the minimum value for the steering axle load was set as 40 kN. In consequence, the Lilliefors test performed again for each SALS confirmed normality of distributions.

### 4.2. Determination and Comparison of Distributions of Means and Standard Deviations of SALS

**Figure 5:**
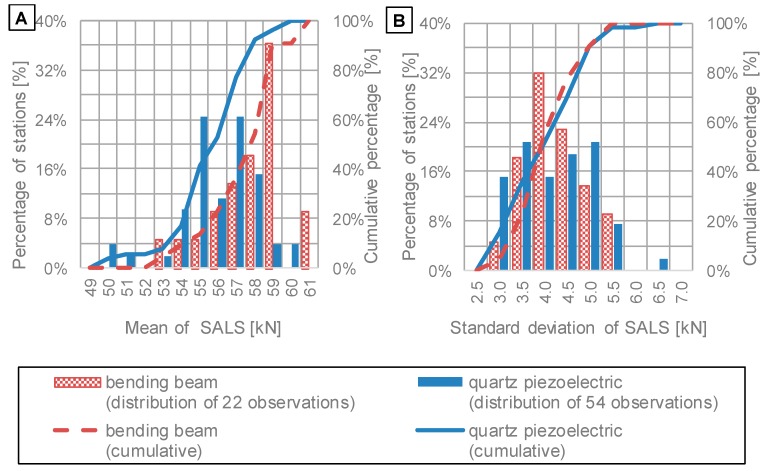
Distributions of (**A**) mean values of SALS and (**B**) standard deviations of SALS calculated on the basis of data from WIM systems equipped with bending beam or quartz piezoelectric axle load sensors.

Normal distributions of SALS were determined for each of the 77 WIM stations; means and standard deviations were used as comprehensive parameters to characterise SALS. It was noted that there were differences, both in mean values and standard deviations of SALS, between particular stations. To characterise the extent of these differences, distributions of previously calculated means and standard deviations were determined. They are shown in Figure 5. Moreover, distributions presented in Figure 5 are divided into two groups of WIM stations, to compare the two technologies of axle load sensors: bending beam and quartz piezoelectric.

Distribution of mean values of SALS, shown in Figure 5A, leads to the following findings:

1. Mean values of SALS range from 50 kN to 61 kN, which implies that systematic error does occur. The range of the observed means is wider in the case of quartz piezoelectric sensors, which implies that this technology of axle load sensors for WIM systems can be potentially more susceptible to relative errors caused by de-calibration.
2. SALS determined from WIM stations equipped with bending beam sensors tend to provide higher values of measured axle loads than WIM stations equipped with quartz piezoelectric sensors. This observation implies that the type of axle load sensor does have an impact on results of measurements of axle loads.
3. It is worth mentioning that stations are maintained by five different companies. Each company performs calibration of WIM stations periodically. In spite of the fact that the same standards are used, some differences in calibration process can occur. Thus, it can be one of the reasons for differences in mean values of SALS.
4. Distributions of standard deviation of SALS, which are presented in Figure 5B, are very similar for both sensor types. This observation leads to the statement that both technologies of axle load sensors are prone to random error to a similar degree. However, the range of standard deviations varies from 3.0 kN to 5.5 kN (up to 6.5 kN in one case), which implies that axle load measurements from a number of WIM stations include higher random error than from other stations.

### 4.3. Investigation of the Sources and Scale of Axle Load Measurement Inaccuracy on the Basis of Mean and Standard Deviations of SALS

**Figure 6:**
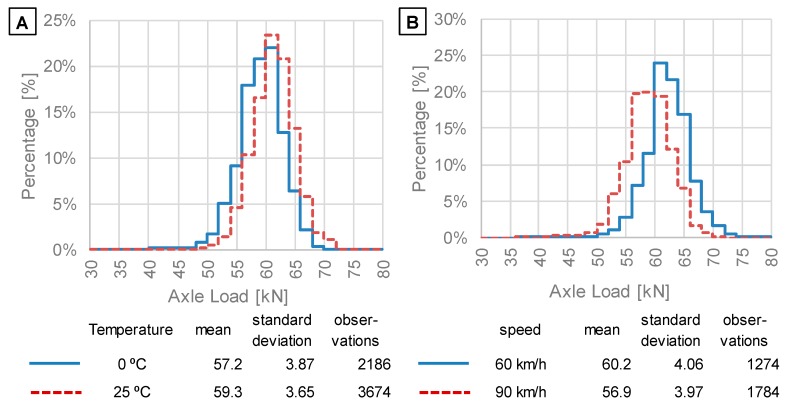
Example of the effect of bias of SALS determined for data from WIM station no. 58 and caused by: (**A**) differences in air temperatures; (**B**) differences in vehicle speed.

Vehicle speed and pavement temperature can be the source of visible differences in means and standard deviations of SALS presented in Figure 5. Therefore, in the next stage of the analysis, SALS were considered for selected intervals of air temperature (with gradation of 5 °C) and intervals of vehicle speed (with gradation of 10 km/h). Figure 6 presents an example comparison of SALS determined for measurements performed at two different air temperatures (Figure 6A) and at two different vehicle speeds (Figure 6B). The four presented SALS were calculated for data delivered from the same WIM station (no. 58). It is visible that an increase in air temperature caused positive bias in SALS. In contrast, an increase in vehicle speed caused negative bias in SALS. The observations from this station are consistent with the theoretical explanation of the phenomena developed in previous research [32,33]. The source of the error is identified as the difference in the distribution of vertical and horizontal stresses in the pavement structure at various conditions of load (speed, temperature). Differences in stress distribution result—to a major extent—from the stiffness moduli of asphalt layers, whose values significantly depend on temperature and vehicle speed.

**Figure 7:**
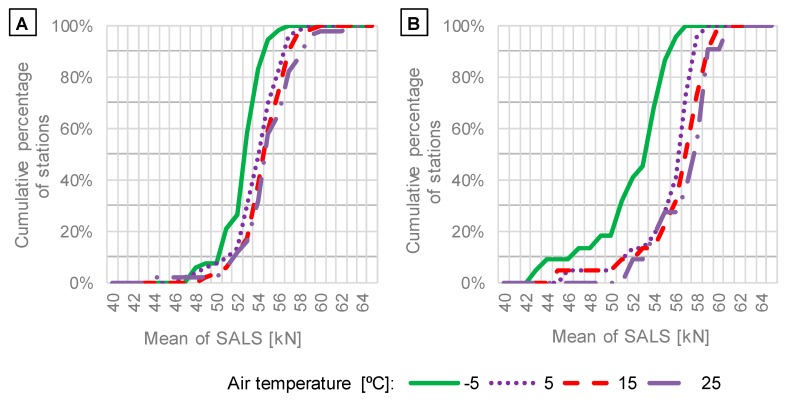
Distributions of mean values of SALS in relation to air temperature based on data from WIM systems equipped with: (**A**) quartz piezoelectric; (**B**) bending beam axle load sensors.

**Figure 8:**
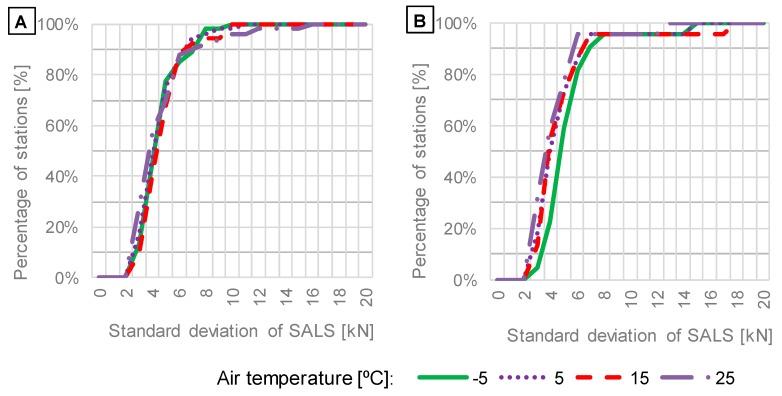
Distributions of standard deviations of SALS in relation to air temperature based on data from WIM systems equipped with: (**A**) quartz piezoelectric; (**B**) bending beam axle load sensors.

**Figure 9:**
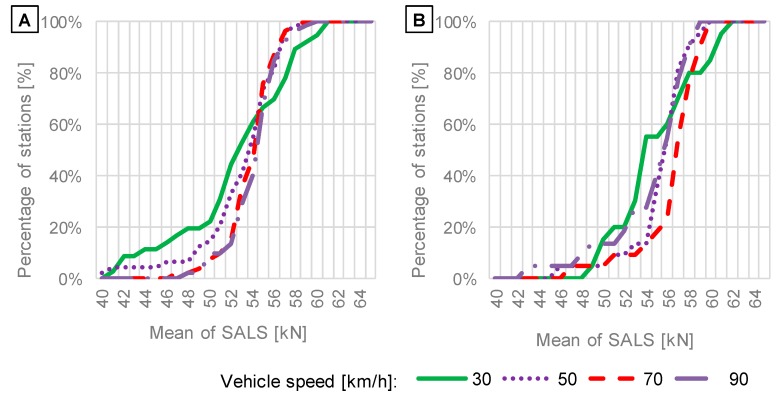
Distributions of means of SALS in relation to vehicle speed based on data from WIM systems equipped with: (**A**) quartz piezoelectric; (**B**) bending beam axle load sensors.

**Figure 10:**
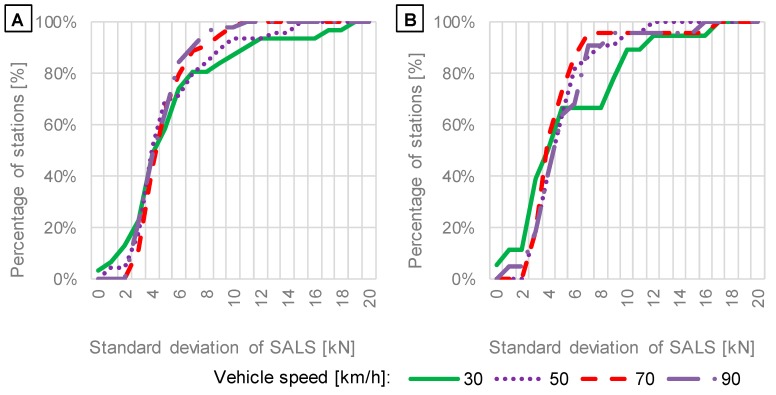
Distributions of standard deviations of SALS in relation to vehicle speed based on data from WIM systems equipped with: (**A**) quartz piezoelectric; (**B**) bending beam axle load sensors.

While the sensitivity of the WIM system to temperature and vehicle speed was detected for site no. 58, not all the considered WIM stations exhibited such visible effects. Due to this fact, a comparison of means and standard deviations of SALS was performed. SALS were calculated again at different temperatures and vehicle speeds for data obtained from WIM stations with particular sensor types according to the procedure showed in Figure 2 in methodology section. The comparison is presented in Figure 7, Figure 8, Figure 9 and Figure 10 as cumulative distributions analogous to those presented in Figure 5. While Figure 6 present SALS and calculated mean and standard deviation for one example of WIM stations, Figure 7, Figure 8, Figure 9 and Figure 10 show means and standard deviations for all of 77 WIM stations.

The effect of temperature on cumulative distributions of mean values and standard deviations of SALS is presented in Figure 7 and Figure 8, respectively.

The investigation of the impact of temperature on means and standard deviations of SALS (Figure 7 and Figure 8) leads to the following findings:

1. The parallel shift of cumulative distributions of mean values of SALS, which is visible in Figure 7, shows that a change in temperature is one of the sources of systematic error. A decrease in temperature causes negative bias of SALS. Bending beam sensors exhibit significantly lower mean values of SALS for the temperature of −5 °C, at which the value is around 10% lower than at the temperature of 25 °C. The effect is also visible in the case of quartz piezoelectric sensors, but the relative difference between means at −5 °C and 25 °C is lower and equals around 5%. This finding is significant when ALS are determined for individual months or seasons, like in input data for MEPDG. The systematic error could be the reason of the decrease in axle loads commonly observed in winter months.
2. According to Figure 8, a change in temperature affects standard deviation of SALS to a minor degree. However, in the case of bending beam sensors, a slight increase in standard deviation is observed for the low temperature of −5 °C. The observations presented in Figure 7 and Figure 8 indicate that the accuracy of bending beam sensors decreases in winter due to an increase in systematic and random error.

The effect of vehicle speed on cumulative distributions of mean values and standard deviations of SALS is presented in Figure 9 and Figure 10, respectively.

The investigation of the impact of vehicle speed on means and standard deviations of SALS (Figure 9 and Figure 10) leads to the following findings:

1. The shapes of cumulative distributions of mean values of SALS (Figure 9) differ from each other, implying that systematic error on WIM systems is sensitive to vehicle speed, and the error increases significantly for the vehicle speed of 30 km/h. For around 60% of stations, mean values at the speed of 30 km/h are lower than means obtained at other speeds, which suggests that axle loads can be underestimated. In contrast, the remaining 40% of stations delivered higher means of SALS, which reflects overestimation of axle loads measured at the vehicle speed of 30 km/h.
2. For typical vehicle speeds of 70 km/h and 90 km/h the cumulative distributions obtained for standard deviations are almost identical. For low speeds, an increase in standard deviations is observed for 25% and 35% of the total amount of WIM stations equipped with quartz piezoelectric and bending beam sensors, respectively (see Figure 10). It shows that in some cases the accuracy can decrease at lower speeds while it is satisfactory at higher speeds. It is noteworthy that the current calibration procedure does not include running of test vehicles at speeds of 30 km/h and lower. The observation presented in Figure 10 is contrary to the hypothesis that random error should increase with vehicle speed due to an increase in dynamic loads. For 60% of WIM stations the standard deviation of SALS equals 5 kN or less, regardless of vehicle speed. However, WIM stations are localised at very smooth road sections. If pavement roughness significantly increased, the random error would probably increase too.

The results delivered from the presented analysis were compared to results from previous works [31,37] and the comparison is summarized in Table 2. Systematic error of WIM measurement can be the result of temperature change, which has been proved both in this study and previous works. However, in some cases of WIM stations the decrease in accuracy can be more problematic than previous studies showed. Moreover, this study revealed that WIM with bending beam sensors underestimate axle load when temperature drops below 0 ℃. Findings delivered from this work converge with previous works on the minor impact of traffic speed on WIM accuracy when vehicle speed is in the range from 50 to 90 km/h. The presented studies showed that when vehicle speed decreased to 30 km/h the WIM systems lost their accuracy regardless of sensor type. Findings presented in these case studies have a theoretical justification [32], that the loss of WIM system accuracy due to change of temperature and vehicle speed rise up from the change of mechanical properties of pavement structure.

### 4.4. Determination of Standard Steering Axle Load Spectrum

The performed analysis of SALS enabled determination of a standard steering axle load spectrum that can be potentially used in algorithms for self-calibration of WIM systems. For this purpose, cumulative distributions presented in Figure 7, Figure 8, Figure 9 and Figure 10 were used. At the first stage, cumulative distributions were used to identify those conditions (air temperatures or vehicle speeds) at which systematic or random error significantly increased. Thus, results obtained for the temperature of −5 ℃ and vehicle speeds of 30 km/h and 50 km/h were excluded. Furthermore, 20% of WIM stations that delivered the lowest mean values and 20% of WIM stations with the highest mean values of SALS were excluded.

The range of mean values determined according to this approach equals from 52 kN to 56 kN for systems with quartz piezoelectric sensors and from 54 kN to 58 kN for systems with bending beam sensors. Mean values of SALS are shifted depending on the type of axle load sensor, thus 55 kN, being the mean value for the range of 52 kN to 58 kN, seems to be the most reasonable value to represent the mean of the standard steering axle load spectrum. Analogously, the range of standard deviations of SALS equals from 3 kN to 5 kN, and the mean for such a range equals 4 kN.

Therefore, definition of the standard steering axle load spectrum as a normal distribution with the mean equal to 55 kN and standard deviation equal to 4 kN is proposed.

## 5. Summary

1. The presented analysis was based on data delivered from 77 weigh-in-motion stations, which are in normal service on motorways and national roads in Poland. Each of the considered weigh-in-motion systems used one of the two axle load sensor technology: bending beam or quartz piezoelectric. The weigh-in-motion data delivered steering axle load spectra, which were further used to investigate the source and scale of inaccuracy of in axle load measurements.
2. In order to include several factors—such as the type of axle load sensor, pavement temperature and vehicle speed—various cases of steering axle load spectra were calculated from a data set selected using a series of filters. The Lilliefors statistical test proved that all the spectra were normal, which enabled valid comparisons of their means and standard deviations.
3. The results of the analysis indicated evident effect of axle load sensor type on the measured axle load. Weigh-in-motion stations equipped with bending beam load sensors tend to provide higher values of axle loads than WIM stations equipped with quartz piezoelectric sensors. Moreover, systems with bending beam sensors are more sensitive to low temperatures, while systems with quartz piezoelectric sensors are more sensitive to changes in vehicle speed, which is expressed by an increase in systematic error. Both technologies are prone to random error to a similar degree.
4. Systematic error of weigh-in-motion systems increases during winter, causing underestimation of axle loads. Systems with bending beam sensors are more susceptible to this problem. When air temperature decreases from 25 ℃ to −5 ℃, the negative bias of axle load spectra equals 5% and 10% for quartz piezoelectric and bending beam load sensors, respectively.
5. Vehicle speed has an impact on the accuracy of weigh-in-motion systems. The increase in error—both systematic and random—is most evident for low speeds (30 km/h). For 25% to 35% of the cases, the random error increases at lower speeds, while it remains at a constant level at higher speeds of 50 km/h to 90 km/h.
6. As a final output of the analysis, a standard steering axle load spectrum was determined. It is a normal distribution with the mean equal to 55 kN and standard deviation equal to 4 kN. It represents a model of steering axle load spectrum obtained for vehicles classified as five-axle articulated vehicles: trucks with two single axles (steering and drive) with one tridem axle in semitrailer. The gross weight of those vehicles is limited to a range of 16 Mg to 20 Mg. The standard spectrum was determined for Polish heavy traffic conditions, which are comparable to conditions in other European Union countries. Nevertheless, the same methodology may be freely adopted in determination of standard steering axle load spectra in other countries.
7. The standard steering axle load spectrum correspond to real traffic conditions, regardless of sensor type, pavement structure, thermal conditions and vehicle speed. In further works the standard steering axle load spectrum will be used to improve self-calibration algorithms for direct enforcement weigh-in-motion systems and also to develop the procedures of traffic data processing for mechanistic-empirical pavement design.

## Figures and Tables

**Table 1:** List of WIM stations considered in the analysis and number of compound data records.

Station ID	Route Number	Latitude and Longitude	Direction (City)	Load Sensor	Number of Records
1	DK3	50.86624 N, 15.61059 E	Jelenia Gora	piezoquartz	4156
2	DK3	50.87413 N, 15.61059 E	Jakuszyce	piezoquartz	2632
3	S7	54.31944 N, 18.73741 E	Warszawa	piezoquartz	16,394
4	S7	54.29416 N, 18.82251 E	Gdansk	piezoquartz	16,248
5	DK7	50.39977 N, 20.09507 E	Krakow	piezoquartz	12,823
6	DK7	50.37171 N, 20.04542 E	Kielce	piezoquartz	9979
7	DK8	50.42416 N, 16.25690 E	Klodzko	piezoquartz	23,278
8	DK8	50.41079 N, 16.27140 E	Kudowa	piezoquartz	24,924
9	DK22	53.68499 N, 17.42860 E	Elblag	piezoquartz	12,025
10	DK22	53.68245 N, 17.53470 E	Gorzow Wlkp.	piezoquartz	13,166
11	DK33	50.42523 N, 16.65970 E	Bystrzyca Klodzka	piezoquartz	2113
12	DK33	50.39948 N, 16.66011 E	Klodzko	piezoquartz	2152
13	DK46	50.69162 N, 18.23691 E	Czestochowa	piezoquartz	7193
14	DK46	50.70081 N, 18.25349 E	Opole	piezoquartz	9177
15	DK75	49.67551 N, 20.66346 E	Brzesko	piezoquartz	8531
16	DK75	49.71107 N, 20.64266 E	Nowy Sacz	piezoquartz	7372
17	DK79	50.11589 N, 19.69721 E	Krakow	piezoquartz	6184
18	DK79	50.11325 N, 19.73722 E	Chrzanow	piezoquartz	4855
19	DK94	50.54573 N, 18.23520 E	Opole	piezoquartz	9492
20	DK94	50.55762 N, 18.20580 E	Strzelce Opolskie	piezoquartz	8991
21	DK19	52.40723N, 22.87044 E	Bialystok	piezoquartz	11,740
22	DK61	53.45242 N, 22.18856 E	Lomza	piezoquartz	48,869
23	DK61	53.42590 N, 22.16835 E	Augustow	piezoquartz	12,170
24	S6	54.44774 N, 17.08001 E	Koszalin	piezoquartz	7588
25	S6	54.42500 N, 17.01472 E	Gdynia	piezoquartz	5042
26	DK91	53.84919 N, 18.80967 E	Tczew	piezoquartz	8937
27	DK12	51.16373 N, 23.03384 E	Chelm	piezoquartz	9376
28	DK12	51.16642 N, 23.06656 E	Piaski	piezoquartz	9927
29	DK19	50.86038 N, 22.28020 E	Janow Lubelski	piezoquartz	6383
30	DK19	50.84445 N, 22.29453 E	Krasnik	piezoquartz	11,469
31	DK17	50.76617 N, 23.22921 E	Krasnystaw	piezoquartz	16,272
32	DK2	52.03615 N, 22.92944 E	Miedzyrzecz Pod.	piezoquartz	18,911
33	DK2	52.03129 N, 22.89799 E	Biala Podlaska	piezoquartz	18,529
34	DK94	50.69450 N, 17.87128 E	Brzeg	piezoquartz	12,323
35	DK94	50.69450 N, 17.87128 E	Strzelce Opolskie	piezoquartz	13,509
36	DK4	49.98664 N, 21.30347 E	Rzeszow	bending beam	21,468
37	DK4	50.00657 N, 21.33720 E	Krakow	bending beam	35,761
38	DK9	50.16616 N, 21.94198 E	Radom	bending beam	22,217
39	DK19	49.54055 N, 21.69250 E	Barwinek	bending beam	15,976
40	DK19	49.49879 N, 21.70061 E	Rzeszow	bending beam	14,789
41	DK9	50.19934 N, 21.88373 E	Rzeszow	bending beam	8963
42	DK77	50.47646 N, 22.23083 E	Przemysl	bending beam	6514
43	DK77	50.44843 N, 22.25040 E	Nisko	bending beam	5007
44	DK92	52.25508 N, 15.44278 E	Swiecko	bending beam	37,131
45	DK92	52.25508 N, 15.44278 E	Swiebodzin	bending beam	14,224
46	DK15	52.83048 N, 18.31217 E	Torun	bending beam	11,398
47	DK15	52.96479 N, 18.53701 E	Poznan	bending beam	19,176
48	S7	51.79546 N, 20.89115 E	Warszawa	piezoquartz	13,331
49	DK2	52.21028 N, 21.82834 E	Siedlce	piezoquartz	11,308
50	DK2	52.20808 N, 21.91044 E	Warszawa	piezoquartz	11,178
51	S11	52.39122 N, 16.74323 E	Poznan	piezoquartz	25,582
52	S11	52.35685 N, 16.76726 E	Pila	piezoquartz	17,475
53	S5	52.51238 N, 17.48577 E	Poznan	piezoquartz	6713
54	S5	52.47277 N, 17.40913 E	Gniezno	piezoquartz	14,198
55	DK11	52.57813 N, 16.82891 E	Poznan	bending beam	17,757
56	S11c	52.52480 N, 16.83285 E	Pila	bending beam	13,715
57	DK11	52.12215 N, 17.38571 E	Jarocin	bending beam	29,343
58	DK11	52.08284 N, 17.40425 E	Poznan	bending beam	24,720
59	DK32	52.22279 N, 16.51789 E	Steszew	bending beam	10,343
60	DK32	52.22557 N, 16.58810 E	Grodzisk Wlkp.	bending beam	15,409
61	DK10	53.18511 N, 16.76446 E	Bydgoszcz	bending beam	21,009
62	DK10	53.15213 N, 16.78864 E	Szczecin	bending beam	19,823
63	DK10	52.88853 N, 19.40468 E	Warszawa	bending beam	15,356
64	DK10	52.88854 N, 19.40506 E	Torun	bending beam	17,909
65	DK15	52.68775 N, 18.20600 E	Poznan	piezoquartz	4607
66	DK15	52.64439 N, 18.18245 E	Bydgoszcz	piezoquartz	14,669
67	DK10	53.16679 N, 17.73500 E	Szczecin	piezoquartz	2583
68	DK10	53.14440N, 17.55297 E	Bydgoszcz	piezoquartz	10,456
69	DK91	53.42240 N, 18.49321 E	Lodz	piezoquartz	14,612
70	DK91	53.27841 N, 18.50998 E	Gdansk	piezoquartz	7108
71	DK45	50.76055 N, 18.02683 E	Opole	piezoquartz	3684
72	DK45	50.75198 N, 18.01024 E	Kluczbork	piezoquartz	3815
73	S3	53.30924 N, 14.58104 E	Gorzow Wlkp.	piezoquartz	9296
74	S3	53.06696 N, 14.86213 E	Gorzow Wlkp.	piezoquartz	11,147
75	S3	53.02968 N, 14.88352 E	Szczecin	piezoquartz	12,940
76	DK10	53.45324 N, 14.37404 E	Szczecin	bending beam	5810
77	S8	51.93614 N, 20.47714 E	Warszawa	piezoquartz	7862
Total number of records					1,019,112

**Table 2:** Comparison of methodologies and results obtained from presented analysis to results obtained from previous works [32,38].

Compared Aspect	This Studies	Studies [31,37]
Approach	Comparison of mean and standard deviations of steering axle load spectra obtained for 77 cases of WIM stations being in normal service.	Comparison of dynamic loads of particular axles with a reference value of axle load for several WIM stations (including MS-WIM).
Effect of temperature	Increase in air temperature from −5 °C to +25 °C causes decrease of mean values of SALS by around 10% in the case of bending beam sensors 5% in the case of quartz piezoelectric sensors, which indicates the relative error caused by temperature change.	Change of pavement temperature within the range −5 °C to +25 °C produced a weighing error change of 10% in the case of bending beam sensor and 7% in the case of quartz piezoelectric
Effect of vehicle speed	Minor effect of vehicle speed on SALS in the range of speed from 70 to 90 km/h. When speed drops below 50 km/h a deterioration of WIM accuracy is observed. The problem is serious both in the case of WIM systems equipped with bending beam and quartz sensors.	The effect of vehicle speed on weighing results for the same vehicle traveling at speeds of 55 km/h and 85 km/h differed by 4% for quartz sensors and by 1% for bending plate sensors.
